# Water Sorption and Water Solubility of Digitally Fabricated Denture Base Materials

**DOI:** 10.3390/ma18235343

**Published:** 2025-11-27

**Authors:** Josip Vuksic, Ana Pilipovic, Tina Poklepovic Pericic, Josip Kranjcic

**Affiliations:** 1 University of Zagreb, School of Dental Medicine, Gunduliceva 5, 10000 Zagreb, Croatia; jvuksic@sfzg.unizg.hr; 2 University Hospital Dubrava, Av. Gojka Šuška 6, 10000 Zagreb, Croatia; 3 University of Zagreb, Faculty of Mechanical Engineering and Naval Architecture, Ivana Lučića 5, 10000 Zagreb, Croatia; ana.pilipovic@fsb.unizg.hr; 4 Department of Prosthodontics, Study of Dental Medicine, University of Split, School of Medicine, Šoltanska 2, 21000 Split, Croatia; tina.poklepovic.pericic@mefst.hr

**Keywords:** CAD-CAM, denture base, water solubility, water sorption

## Abstract

(1) Background: Digitally fabricated denture base materials are increasingly used in everyday clinical practice, but scientific data on their properties is limited, especially regarding water sorption and water solubility. Water absorbed in denture base material penetrates between the polymer chains, acts as a plasticiser, and negatively affects mechanical properties. Residual monomers and other chemicals not chemically bonded in the polymer matrix can dissolve in water and may cause biological consequences such as irritation and inflammation of the oral mucosa or even allergic reactions. This can also have a deteriorating effect on the materials. Therefore, the aim of this study was to investigate the water sorption and water solubility of digitally fabricated denture base materials. (2) Methods: Six denture base materials were used in this investigation: three subtractively manufactured materials, two additively manufactured materials, and one heat-cured poly(methyl methacrylate) material as a control group. The investigation was carried out according to ISO 20795-1:2013. (3) Results: Water sorption for the control group was 28.02 µg/mm^3^, while all digitally fabricated denture base materials showed statistically significantly lower values, ranging between 20.42 and 23.54 µg/mm^3^, with a statistical difference observed within the subtractively manufactured group. All materials had water sorption values below the upper limit of 32 µg/mm^3^ specified in ISO 20795-1:2013. Water solubility for subtractively manufactured materials ranged between 0.04 and 0.40 µg/mm^3^, with values both higher and lower than the control group, but with no statistical difference. Water solubility for additively manufactured materials was 3.01 and 3.22 µg/mm^3^, which was statistically significantly higher than the control group, and these results exceeded the upper limit of 1.6 µg/mm^3^ specified in ISO 20795-1:2013. (4) Conclusions: Digitally fabricated denture base materials showed improved water sorption compared to the heat-cured material, while water solubility for subtractively manufactured materials was similar to the heat-cured material and poorer for additively manufactured denture base materials, with results that did not meet the minimum requirements set by ISO 20795-1:2013.

## 1. Introduction

With many favourable properties, heat-cured poly(methyl methacrylate) is considered the gold standard in denture base fabrication [1]. However, its limitations have driven the development of alternative fabrication techniques and materials, especially in the era of digital dentistry [2].

Dentistry is significantly impacted by new digital technologies [3,4,5]. Subtractive and additive manufacturing are well established in prosthodontics and complete denture fabrication. Their time-saving potential, ability to achieve higher precision, consistency in production, and the possibility of eliminating some fabrication steps make them increasingly popular in everyday use, and these technologies are advancing rapidly [6].

Among the properties that determine the clinical performance of denture base materials, water sorption and water solubility are particularly important, as they affect the durability and comfort of the denture [6,7,8]. Water sorption and water solubility measure the material’s resistance to surrounding oral fluids and are crucial indices when assessing denture base durability [9,10,11]. Water sorption is the ability of the material to absorb liquids, which leads to volumetric changes and alterations in the material’s properties [12,13]. Volumetric changes in denture base materials can cause ageing of the material, affect the stability of the denture in the patient’s mouth, and result in colour changes [2,13,14,15,16]. Absorbed water penetrates between the polymer chains, forces the polymer chains apart, and acts as a plasticiser [13]. This creates internal stress and crack formation over time, and has a deteriorating effect on mechanical properties [8,12,17,18]. It can also reduce the bond strength with relining materials [19]. As it is a polarised molecule, poly(methyl methacrylate) is prone to water sorption, and water molecules can diffuse into the material [20]. Water solubility is a measure of the amount of chemical substances that can dissolve in water at a specific temperature [13]. It is responsible for material loss during immersion [18]. In general, denture base materials have low water solubility, which is caused by the leaching of soluble additives, unreacted monomers, plasticisers, and unreacted initiators, which can cause soft tissue reactions such as irritation, inflammation, or allergy [13,18,21,22]. Water sorption and water solubility are undesirable properties and should remain as low as possible [12,23].

The degree of conversion of the monomer and residual monomer content are associated with increased water sorption and water solubility [2,24]. Differences in the chemical composition of denture base materials are also contributing factors [2,9]. A lower degree of polymerisation in additively manufactured materials results in higher residual monomer content, which can lead to greater water sorption and water solubility compared with heat-cured PMMA [9,12,25]. Materials used for subtractive manufacturing exhibit less porosity and fewer voids, with a higher degree of monomer conversion and greater cross-linking between polymer chains, all of which should reduce the water sorption of the material [19].

Thermocycling, a process that simulates artificial ageing of the material, increases the water sorption and water solubility of all denture base materials [12,13]. Thermocycling can increase water sorption and water solubility by increasing the distance between polymer chains due to thermal stress [26]. Water sorption is a temperature-dependent process, and increased temperature leads to higher water sorption [27,28].

Material innovations and various experiments have been conducted with the addition of different fillers in PMMA materials and in additively manufactured materials to enhance the mechanical and antimicrobial properties of the material. Recently, the addition of nanofillers has attracted growing interest. The addition of fillers also affects the water sorption and water solubility of the material, but with various results [16,18,29,30,31,32]. It can be observed that adding fillers to PMMA and additively manufactured materials can have different effects on water sorption and water solubility, depending on many factors: the type of filler, filler content, filler size and dispersion in the polymer matrix, filler silanisation, polymeric matrix composition, and degree of conversion [8,18,26]. Also, the type of polymer matrix plays a significant role in water sorption and water solubility due to its hydrophilicity and crosslinking [18,33].

A lower degree of polymerisation and higher monomer content in cold-cured PMMA are responsible for higher water sorption and water solubility compared to heat-cured PMMA [34].

The use of disinfectant gels containing chlorhexidine did not have a significant influence on the water sorption and water solubility of heat-cured PMMA [35]. Surface treatments such as polishing and resin coating can reduce the solubility of the denture base material by acting as a barrier [36,37].

Given the limited literature on the topic and the increasing use of digitally manufactured dentures, the purpose of this study was to investigate the water sorption and water solubility of various digitally fabricated denture base materials.

It is important to examine these properties, as they are essential for understanding other material characteristics and the long-term clinical performance of denture base materials.

This study included several commercially available materials currently used in digital denture fabrication. Both additively and subtractively manufactured materials were included in the investigation, using standardised testing procedures, and compared with heat-cured PMMA material. By including materials routinely employed in clinical practice, the research provides practically relevant data and makes a meaningful contribution to optimising material selection and performance assessment in digitally fabricated denture bases.

The hypothesis is stated as follows: digitally fabricated denture base materials have lower values of water sorption and water solubility compared with the control group.

## 2. Materials and Methods

Six different denture base materials were used in this investigation: five were digitally fabricated and one served as the control group. Three materials were used for subtractive manufacturing: Polident pink CAD/CAM (Polident d.o.o., Volcja Draga, Slovenia), Anaxdent pink blank (Anaxdent GmbH, Stuttgart, Germany), and Ivobase CAD pink (Ivoclar Vivadent, Schaan, Liechtenstein). Two materials were used for additive manufacturing: Freeprint denture (Detax, Ettlingen, Germany) and Imprimo LC denture (Scheu, Iserlohn, Germany). Meliodent Heat Cure (Kulzer, Hanau, Germany), a standard heat-cured poly(methyl methacrylate) (PMMA) material, was used as the control group.

The test specimens were discs with a diameter of 50 ± 1 mm and a thickness of 0.5 ± 0.1 mm, with plane-parallel top and bottom surfaces. For digitally fabricated test specimens, an STL file was prepared, and all processing and post-processing procedures were performed according to the manufacturer’s instructions. An Asiga Max UV (Asiga Europe, Erfurt, Germany) DLP 3D printer was used, with a 62 µm resolution and a 385 nm curing wavelength. The Asiga Flash Cure Box (Asiga Europe, Erfurt, Germany) was used for post-curing for 20 min. For the production of Meliodent Heat Cure test samples, a stainless steel template was prepared and the compression moulding technique with heat curing was performed according to the manufacturer’s instructions. For each material, five test specimens were prepared, making a total of 30.

The investigation was conducted in accordance with the ISO 20795-1:2013 specification [38]. Two ovens (ST-05, Instrumentaria, Zagreb, Croatia, and STF-FR 52, Falc, Treviglio, Italy), two desiccators (Bohemia desiccator, 150 mm diameter, Bohemia Crystal Handelsges, Selb, Germany), silica gel (LLG-Desiccant drying agent, 1–3 mm beads, LLG Labware, Meckenheim, Germany), a slide caliper (Unior Digital Calliper 270A, Unior d.d., Zreče, Slovenia), and an analytical balance (AS 220.R2 PLUS, Radwag, Radom, Poland) were used. The investigation took place in a controlled laboratory environment (23 °C, 43% relative humidity).

The investigation involved conditioning the test specimens, storing them in water, and finally reconditioning them. The first oven was set to 130 ± 5 °C and used to prepare fresh silica gel. The second oven was set to 37 ± 1 °C and used to store samples in the desiccator during testing.

**Figure 1:**
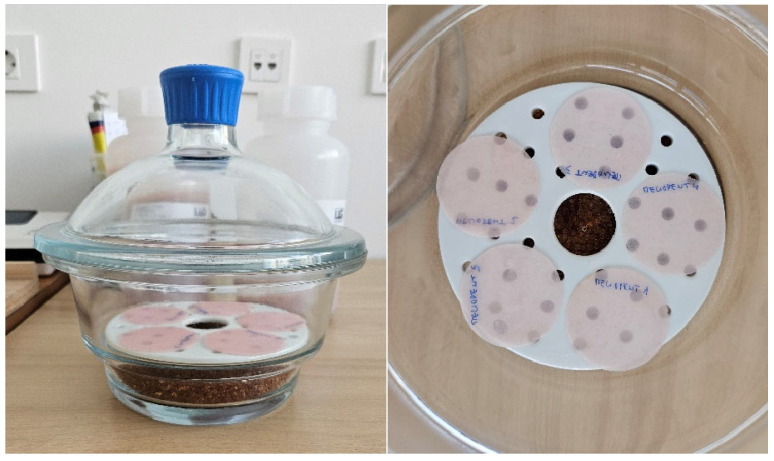
Samples stored in a desiccator with silica gel.

**Figure 2:**
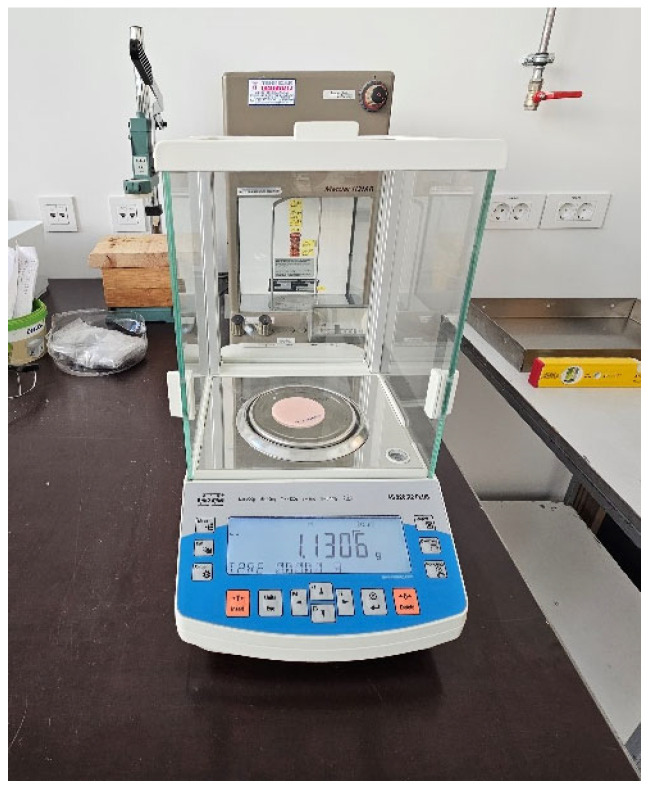
Weighing of the sample with an analytical balance.

For conditioning, samples were stored in a desiccator with freshly prepared silica gel for 23 ± 1 h at 37 ± 1 °C (in the second oven) (Figure 1). Afterwards, samples were transferred to a second desiccator with freshly prepared silica gel. After 60 ± 10 min, samples were ready for weighing using an analytical balance (Figure 2). This cycle was repeated until the difference between successive weighings was less than 0.2 mg, and the conditioned mass *m*_1_ [µg] was recorded. At that point, the volume *V* [mm^3^] of the test specimen was calculated using diameter and thickness measurements taken with a slide calliper.

**Figure 3:**
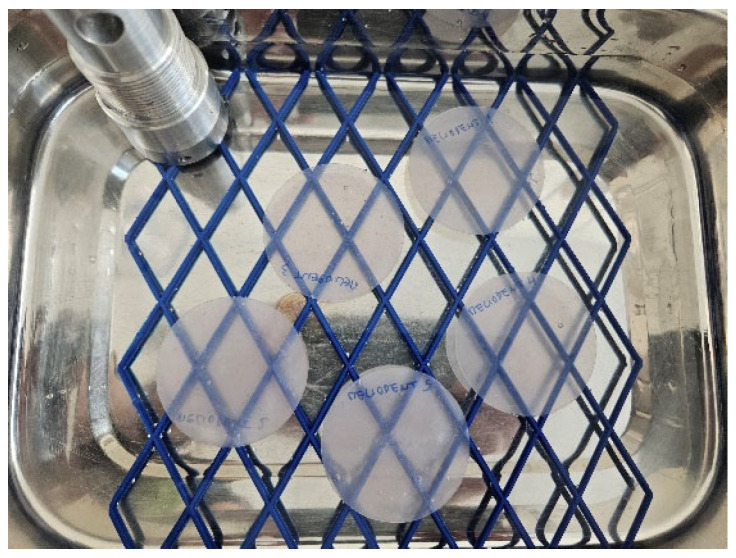
Samples immersed in the water.

After the conditioned mass was obtained and recorded, samples were immersed in water for 7 days ± 2 h in the second oven at 37 ± 1 °C (Figure 3). Next, the samples were removed from the water, wiped with a dry, clean towel, waved in the air for 15 s, and immediately weighed using an analytical balance. The mass *m*_2_ [µg] was then recorded.

Lastly, the samples were reconditioned using the same procedure as for conditioning, and the reconditioned mass *m*_3_ [µg] was recorded at the end.

Water sorption *w_sp_* [µg/mm^3^] was calculated using the following equation:

(1) $$w_{sp}=\frac{m_{2}-m_{3}}{V}$$

Water solubility *w_sl_* [µg/mm^3^] was calculated using the following equation:

(2) $$w_{sl}=\frac{m_{1}-m_{3}}{V}$$

where *w_sp_* [µg/mm^3^] is water sorption, *w_sl_* [µg/mm^3^] is water solubility, *m*_1_ [µg] is conditioned mass of test specimen, *m*_2_ [µg] is mass of the test specimen after immersion in water, *m*_3_ [µg] is reconditioned mass of test specimen and *V* [mm^3^] is volume of the test specimen.

For statistical analysis, IBM SPSS Statistics software v. 29.0.1. was used. In the analysis, a one-way ANOVA test with a Bonferroni post hoc test was used. The Bonferroni post hoc test was used as an addition to one-way ANOVA because this type of post hoc test is especially recommended when testing a single universal null hypothesis that all comparisons are not significant, pertinent in clinical trials or confirmatory analysis settings. Additionally, the Bonferroni post hoc test provides very strong control of the family-wise error rate (FWER), limiting false positives more rigorously than other post hoc tests, especially when the number of comparisons is small to moderate. It is more conservative than Tukey and Holm, substantially lowering Type I error risk. The Bonferroni test also produces narrower confidence intervals for these planned tests, enhancing the ability to detect true differences in this context. *p* values lower than 0.05 were considered statistically significant.

## 3. Results

The obtained data, including conditioned mass *m*_1_, mass after immersion in water *m*_2_, and reconditioned mass *m*_3_ for all materials and specimens, are shown in Table 1.

**Figure 4:**
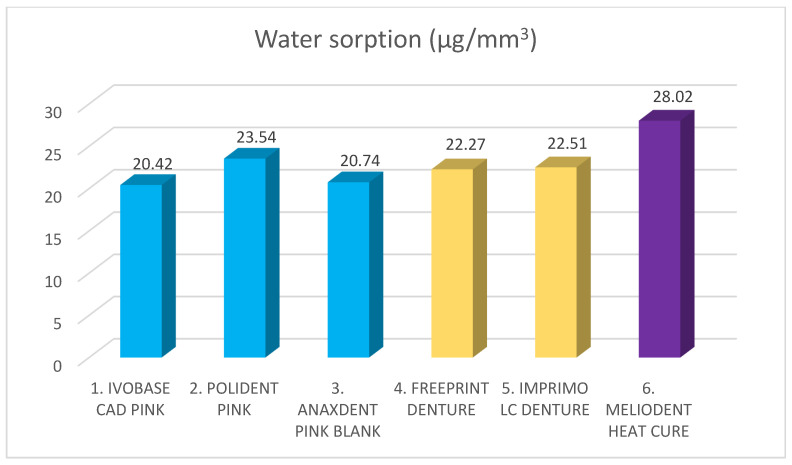
Water sorption of different denture base materials. Blue color represents materials for subtractive manufacturing, yellow color represents materials for additive manufacturing, and purple color represents control group. Mean value stands on top of the bar.

**Figure 5:**
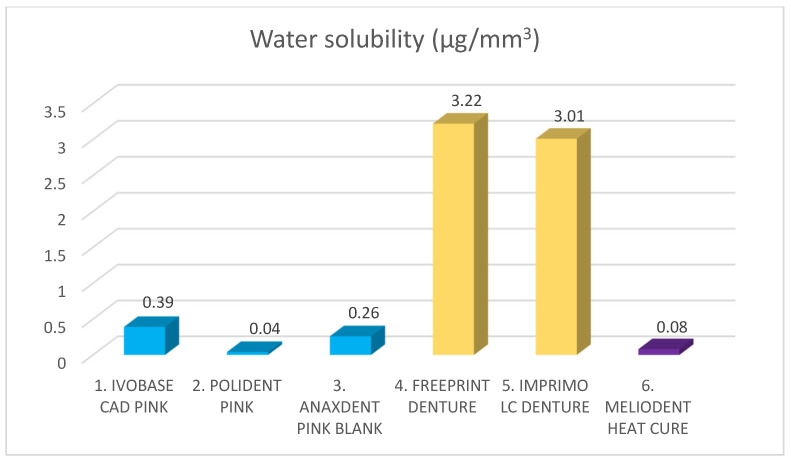
Water solubility of different denture base materials. Blue color represents materials for subtractive manufacturing, yellow color represents materials for additive manufacturing, and purple color represents control group. Mean value stands on top of the bar.

The results of the water sorption and water solubility investigation are shown in Table 2, Figure 4 and Figure 5.

A statistically significant difference was found for both water sorption and water solubility values among different denture base materials (*p* < 0.05). The highest water sorption value was recorded for Meliodent heat cure (28.02 µg/mm^3^), and all other materials showed statistically significantly lower values compared with Meliodent heat cure (*p* < 0.05). There was no statistically significant difference between the two additive manufacturing materials (*p* > 0.05), but a statistically significant difference was observed among for three subtractive manufacturing materials (*p* < 0.05). The lowest water sorption value was found for Ivobase CAD pink (20.42 µg/mm^3^).

The highest water solubility value was recorded for Freeprint denture (3.22 µg/mm^3^). Both additive manufacturing materials showed statistically significantly higher values compared with all other denture base materials (*p* < 0.05). There was no statistically significant difference between the two materials for additive manufacturing and between the three materials for subtractive manufacturing (*p* > 0.05). The lowest water solubility value was found for Polident pink (0.04 µg/mm^3^).

## 4. Discussion

The aim of this study was to evaluate the water sorption and water solubility of digitally fabricated denture base materials using standardised tests described in ISO 20795-1:2013 [38], and to compare these with conventional heat-cured PMMA.

According to the ISO specification, the upper limits for water sorption and water solubility of denture base materials are 32 µg/mm^3^ and 1.6 µg/mm^3^, respectively. For water solubility of cold-polymerised denture base materials, the upper limit is set at 8.0 µg/mm^3^. In this test, all materials met the minimum requirement set by the ISO specification for water sorption and showed values below the upper limit. Additionally, all digitally fabricated materials exhibited statistically significantly lower water sorption values than the control group. However, the water solubility of both additively manufactured denture base materials did not meet the criteria specified in ISO 20795-1, showing values above the upper limit. These results were also statistically significantly higher than those for the control group. Materials produced by subtractive manufacturing met the ISO standard criteria for water solubility, with values both higher and lower than those of the control group, but without statistical significance.

Therefore, the hypothesis was accepted for water sorption values but rejected for water solubility values.

The results of this investigation are partially consistent with previous studies. Altarazi et al. reported statistically significantly higher values for water sorption and water solubility in additively manufactured denture base materials compared with heat-cured PMMA, with all results close to the upper limit set by ISO 20795-1 [6,24]. Gad et al. also found higher values for water sorption and water solubility in additively manufactured materials compared with heat-cured material, with results below the upper limit set by ISO 20795-1 [12]. Perea-Lowery et al. compared additively manufactured materials with heat-cured denture base materials and found similar results for water sorption, but statistically significantly higher values for water solubility in additively manufactured materials [9]. Hada et al. found no statistically significant difference in water sorption and water solubility between heat-cured PMMA and subtractively manufactured material [34].

Gad et al. observed voids in the structure of additively manufactured materials using SEM (scanning electron microscope) [25]. They explained that these voids could originate from microbubbles formed in the resin or could form between layers during printing. In either case, they may contribute to the lower mechanical properties of the material and serve as accumulation points for water, thus increasing the material’s water sorption [13]. Similar findings were reported by Anadioti et al. [39]. In contrast, Altarazi et al. did not find any voids between layers in additively manufactured material that could explain sites for water accumulation [6].

Layer thickness, printing orientation, vat polymerisation technique, post-curing time, and temperature can also influence the properties of additively manufactured material [3,6,7,9,10,40].

The investigation was conducted according to the standard ISO 20795-1:2013. Standard ISO 20795-1:2013 describes standardised methods for measuring water sorption and water solubility; however, it can be observed that some studies used different methods, so comparisons between studies should be made with caution [6,13,15,18,41].

Standard ISO 20795-1 specifies a period of 7 days for immersing samples in water to measure water sorption. A prolonged period of up to 30 days in the study by Dimitrova et al. revealed an increase in water sorption with extended immersion time [13]. Alhotan et al. investigated water sorption and water solubility over a longer period and showed that water gain increased progressively up to day 180, but most water was gained in the first week [18]. Leaching from the material was also highest in the first few days and continued progressively up to day 28. Similar findings were observed in other studies [8,33].

Water is recommended by the standard ISO 20795-1 for storing samples during testing. Using water in this type of testing does not reflect real clinical conditions in which the denture is used, but it allows for the creation of repeatable conditions [29]. Artificial saliva shows similar results to distilled water [18,42]. On the other hand, Zidan et al. showed that immersion in water resulted in higher water sorption and water solubility compared with immersion in artificial saliva [8]. Salivary enzymes can cause degradation of the polymer matrix and negatively influence the properties of the material [43]. Liquids with lower pH values can have a similar effect [44].

Water solubility measures material loss during immersion in water, but questions remain about what is dissolved, what leaks from the material, and in what quantity. This is especially important for materials used in additive manufacturing due to their complex chemical composition and the inclusion of various fillers and nanofillers. Therefore, future research is needed to determine the specific ions and chemical compounds released from the material [29].

## 5. Conclusions

All materials included in this investigation met the minimum requirements set by the ISO standard for water sorption. According to the results, water solubility for both additively manufactured denture base materials did not meet the criteria specified by ISO 20795-1.

All digitally fabricated denture base materials showed statistically significantly lower values of water sorption compared with heat-cured PMMA. Subtractively manufactured materials did not show a statistically significant difference in water solubility values compared with heat-cured PMMA, but additively manufactured materials showed statistically significantly higher values of water solubility compared with heat-cured PMMA and subtractively manufactured materials.

Subtractively manufactured denture base materials showed similar or better results compared with heat-cured PMMA, and, by meeting the minimum requirements set by ISO 20795-1, their clinical use is justified. In contrast, additively manufactured materials showed the poorest results compared with other materials and did not meet the minimum requirements set by the ISO standard for water solubility, so their clinical use is questionable. Further research is necessary to confirm these findings and to determine whether such materials could have a negative impact on patient health.

## Figures and Tables

**Table 1:** Data obtained during the investigation.

	Conditioned Mass *m*_1_	Mass After Immersion *m*_2_	Reconditioned Mass *m*_3_
**Meliodent**			
Sample 1	1.3590	1.3928	1.3588
Sample 2	1.4694	1.5064	1.4696
Sample 3	1.5235	1.5605	1.5235
Sample 4	1.4860	1.5210	1.4856
Sample 5	1.3793	1.4133	1.3792
**Ivobase**			
Sample 1	1.1594	1.1800	1.1588
Sample 2	1.1542	1.1748	1.1539
Sample 3	1.1532	1.1739	1.1529
Sample 4	1.1584	1.1790	1.1579
Sample 5	1.1500	1.1706	1.1497
**Polident**			
Sample 1	1.2159	1.2413	1.2161
Sample 2	1.1854	1.2103	1.1852
Sample 3	1.2401	1.2657	1.2400
Sample 4	1.1832	1.2076	1.1831
Sample 5	1.2236	1.2502	1.2236
**Anaxdent**			
Sample 1	1.1608	1.1820	1.1605
Sample 2	1.1235	1.1448	1.1233
Sample 3	1.1483	1.1680	1.1480
Sample 4	1.1674	1.1896	1.1671
Sample 5	1.1428	1.1626	1.1425
**Freeprint**			
Sample 1	1.4272	1.4525	1.4234
Sample 2	1.3769	1.3989	1.3709
Sample 3	1.4253	1.4493	1.4205
Sample 4	1.4338	1.4598	1.4305
Sample 5	1.4395	1.4657	1.4366
**Imprimo**			
Sample 1	1.0303	1.0493	1.0270
Sample 2	1.0409	1.0603	1.0377
Sample 3	1.0664	1.0863	1.0632
Sample 4	1.0858	1.1058	1.0829
Sample 5	1.1228	1.1435	1.1201

**Table 2:** Water sorption and water solubility results.

Water Solubility [µg/mm^3^]		Water Sorption [µg/mm^3^]		Material
SD	Mean	SD	Mean	
0.14	0.39 ^4,5^	0.05	20.42 ^2,4,5,6^	1. Ivobase cad pink
0.14	0.04 ^4,5^	0.59	23.54 ^1,3,6^	2. Polident pink
0.04	0.26 ^4,5^	1.05	20.74 ^2,4,5,6^	3. Anaxdent pink blank
1.04	3.22 ^1,2,3,6^	0.89	22.27 ^1,3,6^	4. Freeprint denture
0.25	3.01 ^1,2,3,6^	0.52	22.51 ^1,3,6^	5. Imprimo lc denture
0.18	0.08 ^4,5^	0.80	28.02 ^1,2,3,4,5^	6. Meliodent heat cure

SD = standard deviation. Statistically significant difference between materials is indicated with superscripted numbers, *p* < 0.05.

## Data Availability

The original contributions presented in the study are included in the article, further inquiries can be directed to the corresponding author.

## Abbreviations

The following abbreviations are used in this manuscript:

SLA	Stereolithography
CAD-CAM	Computer Aided Design-Computer Aided Manufacturing
ISO	International Organization for Standardization
SEM	Scanning Electron Microscope
DLP	Digital Light Processing
LCD	Liquid Crystal Display
PMMA	Poly(methyl methacrylate)
